# Society Proceedings

**Published:** 1902-11

**Authors:** 


### SOCIETY PROCEEDINGS.

THE forthcoming annual meeting of the Veterinary Medical Association of New Jersey will be held at Trenton, on Thursday, January 8, 1903. One of the most interesting and instructive features of this annual meeting will be a stereopticon lecture by Prof. Veranus A. Moore, of Cornell University, on "Etiology and Prevention of Infectious Diseases of Animals." This will be a very useful and valuable lecture for the practising veterinarian to hear. Dr. Harker, of Trenton, Chairman of the Local Committee of Arrangements, President William Herbert Lowe and Secretary George W. Pope are already arranging and perfecting plans for what promises to be a great and successful State meeting.

### PASSAIC COUNTY VETERINARY MEDICAL ASSOCIATION.

THE regular monthly meeting of the Association was held at 169 Paterson Street, Paterson, N. J., on Tuesday evening, October 7, 1902, with Dr. William Herbert Lowe, President, in the Chair.

The following members were present: Drs. William J. Reagan, T. J. Cooper, H. K. Berry, M. A. Pierce, William Herbert Lowe, of Paterson; John Kehoe, J. Payne Lowe, of Passaic; and William J. Fredericks, of Delawanna.

Dr. H. K. Berry was chosen Secretary pro tem.

The minutes of the previous meeting were read and approved.

Treasurer M. A. Pierce reported that he had received $16 from Secretary Machan; had paid the Guardian Printing and Publishing Company's bill of $9.75 for printing, leaving a balance of $6.25. The Treasurer's report was ordered entered upon the minutes.

President Lowe reported that Dr. Brooks had returned from his vacation and had made application for membership The President stated that Dr. Brooks stood for the advancement of the profession in every way, and was proud of the fact that the movement to organize a local society had the unanimous support of the veterinary practitioners of Passaic County. Dr. Brooks received the unanimous vote of all present, and he was declared a member of the Association.

The President reported that he had ordered a bill of the Guardian Printing and Publishing Company of $9.75 paid. This bill was for printing the Constitution, By-laws, and Code of Ethics of the Association.

Under Article IX. of the By-laws the following proposition was presented to the meeting and carried by a unanimous vote:

In view of the earnest and successful efforts of Senator Wood McKee in the halls of the State Legislature at Trenton last winter, which resulted in the enactment of Chapter XVIII., Laws of 1902, "An act to regulate the practice of veterinary medicine, surgery, and dentistry in the State of New Jersey, to license veterinarians, and to punish persons violating the provisions thereof;

Therefore we, members of the Passaic County Veterinary Medical Association, do hereby propose Hon. Wood McKee for honorary membership in this Association.

William Herbert Lowe,
W. J. Reagan,
William J. Fredericks.

President Lowe declared Senator Wood McKee an honorary member.

Dr. Fredericks moved that the President be authorized to have certificates of membership printed; carried.

Moved by Dr. Kehoe, and seconded by Dr. Cooper, that an invitation be extended to the veterinarians of Bergen County to attend our meetings; carried.

The members listened with much interest to the reading of an excellent paper on "Professional Etiquette" by Dr. J. Payne Lowe, of Passaic. Dr. Lowe's paper was received, discussed, and ordered placed on file.

A vote of thanks was extended to the doctor for the care he had exercised in the preparation of his paper.

Dr. Cooper was appointed essayist for the next meeting, his subject being "The Transmission of Disease Through Milk."

The meeting adjourned at 10.45 P.M.

William Herbert Lowe,
President.

H. K. Berry,
Secretary pro tem.

---

### CONNECTICUT VETERINARY MEDICAL ASSOCIATION.

The semi-annual meeting of the Association was held at "Hotel Hartford," in Hartford, Tuesday, August 5, 1902.

The President, Dr. A. Hyde, being absent, the meeting was called to order by Second Vice-President Dr. Harrison Whitney, of New Haven, at 3 o'clock.

Seven of the thirty-nine members responded to roll-call, viz.: Drs. L. B. Judson, of Winsted; P. F. Finnegan, of Hartford; E. C. Ross, of New Haven; C. R. Witte, of New Britain; Harrison Whitney, of New Haven; B. K. Dow, of Willimantic; George H. Parkinson, of Middletown.

Visitors: Drs. G. W. Loveland, of Torrington, and F. F. Bushnell, of Winsted; also reporters of the Hartford *Times* and *Courant*.

The minutes of the last meeting were read and accepted as written.

It was voted to order twenty-five certificates of membership for the Association, and the Secretary was instructed to procure the same.

It was voted to lay Article IV. of the By-Laws on the table until the annual meeting. The report of the Treasurer showed a balance of $24.

Dr. Frederick F. Bushnell, of Winsted, and Dr. G. W. Loveland, of Torrington, offered their names for membership in the Association. Their names were referred to the Board of Censors for action.

The President, Dr. Andrew Hyde, formerly of Norwich, now employed in the United States Bureau of Animal Industry, and located at Sioux City, Iowa, presented his resignation as President of the Association, which was accepted.

It was voted to hold the annual meeting of the Association at "Hotel Hartford," in Hartford, the first Tuesday in February, 1903, at 3 o'clock.

Dr. Ross suggested that the President select three topics for discussion at the next meeting.

The President selected the following subjects: 1. "Canine Distemper." 2. "Azoturia." 3. "Scrotal Hernia."

The matter of papers for the next meeting was left with the Secretary.

After discussing several cases and matters of general interest to the profession, the meeting adjourned. B. K. Dow, V. S., Secretary.

---

### AMERICAN VETERINARY MEDICAL ASSOCIATION.

### Appeal of Dr. J. G. Rutherford, Chief Veterinary Inspector, for the Convention of 1903 for Ottawa, Canada.

As you are probably aware, an invitation has been extended to the American Veterinary Medical Association to hold its next annual meeting in Ottawa. The idea of having the meeting at the Canadian capital was first introduced by me at the recent gathering in Minneapolis, and has been heartily endorsed not only by the other veterinarians of the city, but by every one else here to whom the matter has been mentioned. The veterinary practitioners at a recent meeting passed the following resolution: "That the Veterinary Practitioners of the City of Ottawa, here assembled, unanimously endorse the invitation extended by Dr. Rutherford to the American Veterinary Medical Association to hold its next annual meeting at Ottawa, and look forward with pleasure to the opportunity which will thus be afforded them of becoming acquainted with the members of that distinguished body and assisting in their entertainment during their visit to the capital of Canada.

The Mayor and Council of the City have forwarded to the Executive a cordial invitation to meet here next year, while the officers of the Department of Agriculture are also prepared to do their share in entertaining the members of the Association. Mr. Edwards, M. P., has expressed a desire that the Association should accept his hospitality for one day by going down the river twenty-four miles to Rockland and inspecting his magnificent herd of Shorthorns, which, for a number of years has been conducted on the "Bang" system, under the supervision of one of our veterinary officers. Another afternoon can be pleasantly and profitably spent at the Central Experimental Farm just outside the city.

There are numerous other attractions, such as the Parliament building, the Chaudiere Falls, the parks at Aylmer, Britannia, and Rockcliffe, etc., which ought to make the visit interesting and agreeable, not only to the the members, but to the ladies accompanying them.

Apart from all these, however, I may say that I am convinced that great good, not only to the Association but to the profession generally, and especially to Canadian practitioners, will result from the holding of this meeting in Ottawa. The influx of such a large body of intelligent, highly trained, and up-to-date veterinarians cannot fail to arouse widespread and most beneficial interest among the members of the profession throughout the Dominion.

That some such awakening is needed there can be no question, and it seems to me that the present is an opportunity which should, on no account, be neglected. I am satisfied, also, that the membership of the Association will be largely increased by the affiliation of many Canadian veterinarians who have hitherto looked upon the American Veterinary Medical Association as a foreign body, and not what it really is, an organization having for its sole object the development and general betterment of the veterinary profession on the North American Continent, regardless of international boundaries or political relations.

In view of all these facts, might I ask of you to use your influence with the members of the Executive in favor of the selection of Ottawa as the next place of meeting? You will all be warmly welcomed.

---

### A. V. M. A. RESIDENT STATE SECRETARIES, 1902–1903.

Alabama: L. Van Es, Mobile.
Arizona and New Mexico: J. C. Norton, Phœnix.
Arkansas: R. R. Dinwiddie, Fayetteville.
British Columbia: Johnson Gibbins, 1003 Granville St., Vancouver.
California: J. J. Summerfield, Santa Rosa.
Colorado and Utah: Thomas Castor, box 525, Trinidad.
Connecticut: Thomas Bland, Waterbury.
Delaware: H. P. Eves, 507 W. Ninth St., Wilmington.
District of Columbia: A. M. Farrington, 1436 Chapin St., Washington.
Florida: J. G. Hill, 324 Forsyth St., Jacksonville.
Hawaiian Islands: W. T. Monsarrat, Honolulu.
Illinois: E. L. Quitman, 489 Jackson Blvd., Chicago.
Indiana: J. R. Mitchell, Evansville.
Iowa: Hal C. Simpson, Denison.
Kansas: N. S. Mayo, Manhattan.
Kentucky: D. A. Piatt, Lexington.
Louisiana: E. Pegram Flower, Baton Rouge.
Manitoba: W. J. Hinman, Winnipeg.
Maryland: L. A. Nolan, Dillon and Fifth Sts., Baltimore.
Massachusetts: Benj. D. Pierce, 27 Sanford St., Springfield.
Michigan: G. W. Dunphy, Quincy.
Minnesota: J. G. Annand, 414 First Ave., S. E., Minneapolis.
Mississippi: J. C. Robert, Agricultural College.
Missouri: T. B. Pote, 4046 Cottage Ave., St. Louis.
Montana: M. E. Knowles, Helena.
Nebraska: H. Jensen, Weeping Water.
New Hampshire and Maine: Lemuel Pope, Jr., 101 State St., Portsmouth.
New Jersey: T. E. Smith, 309 Barrow St., Jersey City.
New York: Wm. Henry Kelly, 233 Western Ave., Albany.
Nevada and Idaho: J. Otis Jacobs, Reno.
North Carolina: A. S. Wheeler, Biltmore.
North and South Dakota: W. F. Crewe, Devil's Lake.
Nova Scotia: Wm. Jakeman, Halifax.

Ohio: A. S. Cooley, 1184 E. Madison Ave., Cleveland.
Ontario: John W. Groves, Hamilton.
Oregon: Wm. McLean, 328 Fourth St., Portland.
Pennsylvania: C. J. Marshall, 2004 Pine St., Philadelphia.
Quebec: Chas. H. Higgins, Department of Agriculture, Ottawa.
Rhode Island: Thos. E. Robinson, 65 Main St., Westerly.
South Carolina and Georgia: G. E. Nesom, Clemson College.
Tennessee: W. C. Rayen, Nashville.
Texas: M. Francis, College Station.
Washington: Clarence Loveberry, per Frye-Bruhn Co., Seattle.
West Virginia: F. P. Ruhl, Fairmont.
Wisconsin: J. T. Hernsheim, Market and Exchange Sts., Kenosha.

